# Implementation of sustainable complex interventions in health care services: the triple C model

**DOI:** 10.1186/s12913-021-06115-x

**Published:** 2021-02-15

**Authors:** Hanan Khalil, Kathryn Kynoch

**Affiliations:** 1 School of Psychology and Public Health, La Trobe University, Level 3, 360 Collins Street, 3000 Melbourne, Vic Australia; 2 Evidence in Practice Unit and The Queensland Centre for Evidence Based Nursing and Midwifery, A JBI Centre of Excellence, Mater Health, Brisbane, Australia; 3 The Queensland Centre for Evidence Based Nursing and Midwifery, A JBI Centre of Excellence, Adelaide, Australia

**Keywords:** Implementation, Health services, Sustainability, Translation

## Abstract

**Background:**

The changing and evolving healthcare environment means organisations are under increasing pressure to deliver value-based, high quality care to patients through enabling access, reducing costs and improving outcomes. These factors result in an increased pressure to deliver efficient and beneficial interventions to improve patient care and support sustainability beyond the scope of the implementation of such interventions. Additionally, the literature highlights the importance of coordination, cooperation and working together across areas is critical to achieving implementation success. This paper discusses the development of a triple C model for implementation that supports sustainability of complex interventions in health care services.

**Methods:**

In order to develop the proposed implementation model, we adapted the formal tradition of theory building that is described in sociology. Firstly, we conducted a review of the literature on complex interventions and the available implementation models used to embed these interventions to identify the key aspects relating to successful implementation. Secondly, we devised a framework that encompassed these findings into a simple and workable model that can be easily embedded into everyday practice. This proposed model uses clear, systemic explanation, adds to the current knowledge in this area and is fit for purpose, providing healthcare workers with a simple easy-to-follow framework to embed practice change.

**Results:**

A three-stage implementation model was devised based on the findings of the literature and named the Triple C model (Consultation, Collaboration and Consolidation). The three stages are interconnected and overlap to support sustainability is considered at all levels of the project ensuring its greater success. This model considers the sustainability within any implementation project. Sustainability of interventions are a key consideration for continuous and successful change in any health care organisation. A set of criteria were developed for each of the three stages to support adaptability and sustainment of interventions are maintained throughout the life of the intervention.

**Conclusion:**

Ensuring sustainability of interventions requires continuing effort and embedding the need for sustainability throughout all stages of an implementation project. The Triple C model offers a new approach for healthcare clinicians to support sustainability of organizational change.

## Background

Given the changing landscape of healthcare in recent years, providing high-quality care through the implementation of evidence-based innovations whilst also reducing costs has become a priority for healthcare organisations [1]. While providing value for the patient is important, in order to achieve value-based healthcare, sustainability of implemented interventions is crucial. In areas identified for improvement, the implementation process needs to be simple with sustainability a key focus throughout. The necessity of coordination, cooperation and working together across areas is critical to achieving success [2]. It is important to consider how the complexities of healthcare influence the implementation process.

## Complex interventions

The high prevalence of chronic conditions, aging populations and new endemics have increased the pressure on health services to incorporate interventions seen as “complex interventions” [3, 4]. These complex interventions often contain a number of interacting components as well as many different outcomes to improve the health care delivery and increase patient safety [5, 6].

Numerous papers report complex interventions that have been integrated into health care practice to improve the safety and quality of care provided to patients [7–12]. The types of interventions range from behavioural, technological, organisational and clinical and include a variety of consumers in health services. An early study by Campbell and colleagues has highlighted the importance of determining the effectiveness of complex interventions in health care and detailed a step-wise approach for the design and evaluation of such interventions [13]. They based their work on the Medical Research Council Framework for the development and evaluation of randomised controlled trials for complex interventions [14]. Moreover, the authors suggested that when planning for implementation of complex interventions, the steps needed to be clearly outlined and include a clear definition and understanding of the problem and its context, describe the development of the intervention and finally optimise the evaluation of the intervention based on three possible scenarios. These scenarios included: a consideration of the cost and resources involved, the evidence supporting the benefits of the intervention and finally the cost benefit ratio of the intervention [14].

## Theoretical models

Many theoretical models have evolved to simplify the implementation of such interventions and provide information about evaluation of these interventions [15–35]. The terms, theories, models and frameworks have been used interchangeably in implementation science literature. However, they are distinct in their definitions. A theory usually provides a clear explanation of how and why a specific phenomenon exists [15]. A model typically involves a deliberate simplification of a phenomenon or a specific aspect of a phenomenon. Models are also sometimes referred to as a narrow form of a theory and are descriptive, as opposed to theory which is explanatory as well as descriptive. Frameworks on the other hand do not provide explanations, they are a set of empirical phenomena that are translated into a set of categories. A framework usually denotes a structure, overview, outline, system or plan related to a specific phenomenon. The current theories, models and frameworks used in implementation science have been summarised by Nilson (2015) in order to make sense of the available methodologies. They all fall under three overarching objectives; guiding the process of translation of research into practice (process models), explaining what influences implementation outcomes (determinant frameworks, classic theories, implementation theories) and evaluating implementation (evaluation frameworks) [15].

Process models for evidence implementation originated mainly in the nursing field [5, 11]. Some examples of known process models include ACE (Academic Center for Evidence Based Practice) Star Model of Knowledge Transformation, the Knowledge to Action Framework, the Iowa Model, the Ottawa Model for Research Use and the Joanna Briggs Institute (JBI) model [18–21]. The focus of these models is on how to implement evidence into practice. Their main emphasis is on careful planning using a stepwise linear approach to implementation as one step usually follows another.

Explanatory frameworks for implementation usually include steps that identify barriers and facilitators to implementation specifically considering the context in which the innovation is being implemented [24–28]. Most of these frameworks rely on either individual or organisational change, climate, culture and leadership. Whereas others are based on specific theories such as behavioural change and social cognition An example of this based on theories is the Theoretical Domains Framework.

Examples of other types of similar frameworks include: Promoting Action on Research Implementation in Health Services (PARIHS), the conceptual model, Ecological framework and Consolidation for Implementation Research (CFIR) [9, 27, 28].

The third type of implementation approach is evaluation frameworks [32–35]. These frameworks provide a structure for evaluating implementation or quality improvement projects. Examples of these include Re-AIM (Reach, Effectiveness, Adoption, Implementation, Maintenance) and PRECEDE-PROCEED (Predisposing, Reinforcing and enabling constructs in Educational Diagnosis and Evaluation-Policy, Regulatory) and Organisational constructs in Educational and Environmental Development [32–35].

In this paper firstly, we will focus on examining and identifying the facilitators and barriers involved with complex interventions and provide an alternate approach - The Triple C model - as a simple easy to use model for clinicians to use to implement interventions and support sustainability in clinical practice. Secondly, we will provide examples demonstrating the successful use of the model across a range of health care settings and areas of practice including; wound care, medication safety, palliative care, oncology and haematology.

## Method

In order to develop the proposed implementation model, we adapted the formal tradition of building a theory that is described in sociology [36–38]. Within sociology, theory is defined as multiple ideas that form the basis of three types of conceptual work; describing, explaining and predicting observed phenomena. We undertook a two-stage process. Firstly, a review of the literature on complex interventions and the available implementation models employed to embed these interventions. The literature search included a search of three databases Medline, Embase and Google Scholar using key terms such as, complex interventions, implementation methods/framework, sustainability and healthcare, barriers and facilitators. Full text of relevant articles were read and data were extracted and summarised in a table to identify the types of interventions, barriers and facilitators encountered by the researchers. We used a scoping review methodology where the subjects of the review were complex interventions, the concept was the barriers and facilitators of implementing them and the context was the health care setting [39, 40]. A data extraction of these main characteristics was devised and presented in Table 1.

**Table 1:** Barriers and facilitators of project implementation

Study	Country	Interventions	Barriers	Facilitators
Bach-Mortensen 2018 [41]	UK	Evidence based interventions	Organisational culture Lack of Support and expertise	Engagement of central stakeholders, funders, clinicians
Barnett 2011 [42]	UK	Health care innovations	Lack of quantitative evidence The influence of human-based resources the impact of organisational culture and resources	Interorganisational partnership
Bird 2014 [43]	UK	Complex mental health interventions	Lack of staff skills to deliver the intervention Complexity of intervention Time constraints Lack of reimbursements and incentives	Ongoing support and supervision Relevance to organisational culture and values Cost benefit ratio
Bergs 2015 [44]	Belgium	Surgical safety checks	Workflow adjustments as proposed by organisational structure Staff perception	Good leadership Relevance of intervention and local context
Colvin 2013 [45]	South Africa	Task shifting interventions	Lack of evidence about the intervention Lack of training, supervision and support	Teamwork
Ling 2012 [46]	UK	Integrating care	Organisational structure Lack of Information technology Financial arrangement Governance	Staff Involvement and support Relationship between leaders
Humphries 2014 [47]	Canada	In program management	Organisational structure and process Organisational culture	Successful individual interaction with others in the organisation
Kormelinck 2020 [48]	Netherlands	complex interventions for residents with dementia	Communication and coordination between disciplines Lack of Management support Unstable organisations High staff turnover Perceived work and time pressure	Sufficient resources Openness to change (Organisational culture) Strong leadership and support of champions
McGinn 2011 [49]	Canada	Electronic health care records implementation	Lack of time and workload proposed by the organisational structure The degree of difficulty of the interventions	Patient and health professional interaction
Pescheny 2018 [50]	UK	Social prescribing services	legal agreements staff turnover, staff engagement, Lack of infrastructure provided by the organisations	Positive leadership and management Relationships and communication between partners and stakeholders
Verberne 2018 [51]	Netherlands	Paediatric Palliative care interventions	Lack of clarity of tasks provided by leaders within the organisation	The simplicity and clarity of the intervention The recognition of the need of the intervention
Vlaeyen 2017 [52]	Belgium	Fall prevention interventions	Limited knowledge and skills Staffing issues Poor management Poor communication	Good communication Availability of resources
Wood 2017 [53]	UK	Collaborative care addressing depression interventions	Lack of role clarity	improving inter-professional communication

 Secondly, a framework was devised that encompassed the findings from the scoping review into a simple and workable model that can easily be embedded into everyday practice and yet fit the criteria of a theory. We used a formal theory building used in sociology [54–56]. This framework was successfully used by other authors such as May et al., 2007 to build theories. It allows researchers to evaluate the generalisability of the framework to other settings and support congruence and reliability in different situations [5, 6]. The criteria upon which the theory were built was as follows.

1. It must have accurate description - this refers to the clarity of definitions used in the model,
2. It must have systematic explanation-this refers to the provision of enough explanation of the phenomenon in form of causal or relational mechanisms,
3. It must support knowledge claims- this refers to the use of theory in resulting knowledge claims in the forms of explanations, analytical propositions or experimental hypothesis and.
4. It must be testable - this refers to that fact that theory must be tested to support its concrete and fit for purpose.

**Fig. 1:**
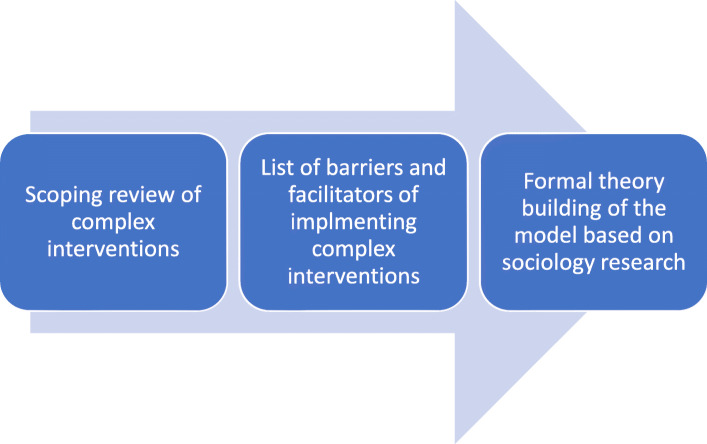
Model development of the Triple C

The above framework was used to devise the components of the model that explains the successful implementation of complex interventions in healthcare as shown in Fig. 1.

## Results

### Barriers and facilitators of complex interventions in health services

A total of thirteen studies were identified from our search. Several factors have been identified in the literature as barriers and facilitators for implementing complex interventions as shown in Table 1 [41–53]. Organisational barriers include organisational culture, support from leadership and the availability of resources. Other common barriers across the studies included: education and training needs of staff, time constraints, complexity of intervention, lack of staff engagement and poor management and communication. Facilitators for implementing interventions included: sufficient resources, engagement of stakeholders, staff involvement and support from leaders and staff. Staffing issues were commonly cited barriers and facilitators than type of intervention [43–45, 48–50, 53]. The complexity of intervention was only cited in a few studies [41–53].

Staffing issues are by far the most complex to address when implementing interventions in health services as opposed to organisational factors [57]. Several key factors were identified from the literature. Understanding human behaviour, decision-making during critical situations and identifying sources of errors are key considerations and have the potential to influence the success or failure of project implementation. Moreover, effective teamwork requires cooperation, coordination and communication between the various team members [58]. Effective communication between and within teams enables cooperation and coordination. To support success every member of the team requires an understanding of the purpose, team roles, responsibilities, task requirements and the project plan. Trust in other team members and sharing information are also essential to enable cooperation between teams [57–61].

The synthesised model of implementation (the Triple C model) builds on these key staffing issues to enable successful implementation of complex interventions in health services as described in the next section [62].

## Discussion

### The development of the triple C model

The Triple C model proposes that to achieve successful implementation in health services requires attention to the social relations and processes that will result in outcomes.

**Fig. 2:**
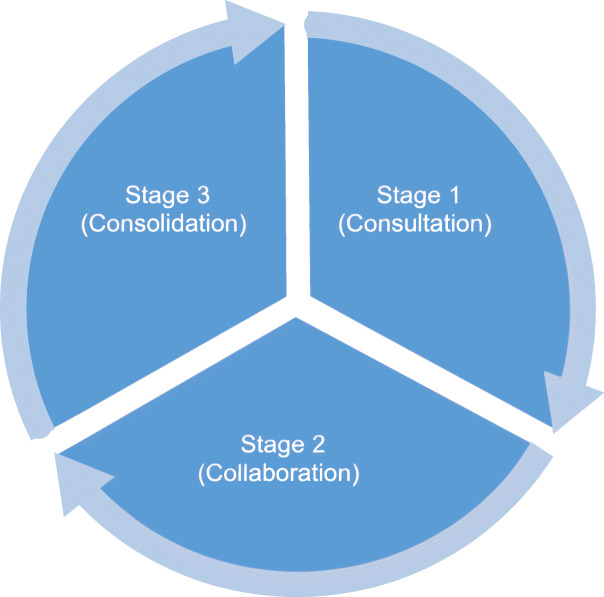
The Triple C model (Consultation, Collaboration and Consolidation)

It emphasises the processes by which complex interventions can be made practicable and embedded into daily clinical care by underscoring the significance of staffing issues. A three-stage implementation model was devised based on the findings of the literature and named the Triple C model (Consultation, Collaboration and Consolidation) as shown in Fig. 2. The three stages of the model are in interconnected and overlap. As opposed to other models, this model incorporates the consideration of sustainability at all stages of the implementation project.

Sustainability of interventions are a key factor for continuous and successful change and can lead to reduction in resistance to change and a shift in organisational culture. Examples of sustainability strategies include: long-term action plans, tracking of program adoption, financial planning and mapping of the community settings where interventions take place. Sustainability has been defined in the literature as routinization, institutionalisation, durability, maintenance and long-term follow-up of an implementation [10, 34, 57, 58]. Stirman and colleagues suggested that for an intervention to be sustainable certain core elements of the initial intervention must be displayed and maintained after the initial implementation of the intervention [63]. Moreover, most researchers have conceptualised implementation to be the last step of any implementation process. The Triple C model conceptualises sustainability as a set of processes that occur throughout the life cycle of any implementation process. The continuous consultation, collaboration and consolidation supports that sustainability is not an end point but is a continuous process whereby the three stages are interconnected and overlap with each other to achieve sustainment [62].

Furthermore, health service research studies have increasingly recognised the value of adaptation in light of the everchanging context of health care services. Adaptability of an intervention to local context is necessary to support the usability and relevance of such interventions [64]. The relationship between adaptability and sustainability has been discussed in depth by Shelton et al., 2018. Shelton and colleagues highlighted the importance of identifying barriers and facilitators to adaptability of an intervention to support its sustainability [10]. This is in addition to ensuring the core elements of the initial intervention are still maintained. The Triple C model allows for adaptability and sustainability through the continuous engagement of project stakeholders.

### The consultation stage

The consultation stage is typically the initial stage of any implementation model and this is where all stakeholders can prioritise their workflow and initiate ideas and suggest areas for improvement. This stage should capture all the stakeholders’ priorities and map the pathway that will be taken to support the successful implementation of the project. A process map of the key steps involved in the project supports a clear pathway of the project trajectory, areas for improvement and monitoring as shown in Table 2. To date, process mapping has not been used extensively in health care [60]. A study by Antonacci et al., 2018 highlighted the advantages of using process mapping for planning projects in health care [11]. The authors highlighted five key factors for successful process mapping including: appropriate and easy visual representation of the project; information collected from stakeholders; the ability of the facilitator to gather ideas from those involved and capture them on the map; knowledge of software and equipment used if needed and the ability to follow-up any missing steps or information throughout the process.

**Table 2:** The components of the Triple C model

Stages of the Triple C model	Enablers
**Consultation**	• Prioritising of ideas • Identification of areas of improvement • Design of a process map
**Collaboration**	• Clarity around roles and responsibilities • Understanding of organisational change • Culture of the organisation
**Consolidation**	• Standardising policies and protocols • Eliminating variances between policies and practice • Right staff mix with appropriate skills • Knowledge of patients’numbers • Sufficient resources • Business Intelligence tools

### The collaboration stage

The collaboration stage aims at identifying who should be involved in the project based on their skills, knowledge and contribution to the overall project. This stage requires a high level of communication and openness between team members. Nystrom et al., 2018 highlighted the importance of collaboration on health projects from an interdisciplinary perspective to support the success of the project [12]. Several collaborative approaches can be used for successful implementation. These methods range from including higher degree students in the projects, clinicians having dual roles in the project as researchers and clinicians and involving staff from various levels of healthcare [12]. The challenges of successful collaboration include lack of clarity around roles and responsibilities in the project plan, organisational changes such as staff turnover, changing of policies or priorities and cultural differences amongst the project team [43, 51–53]. On the other hand, enablers of good collaboration include established relationships, alignment of goals and priorities, skilled team members, clear communication, mutual trust and honesty between team members as shown in Table 2 [41, 46, 52, 53].

### The consolidation stage

This stage is the most crucial step in the model as it supports the sustainability of the project and its incorporation into routine clinical care [3, 24]. This stage may involve refinement of the initial ideas to support their successful adaptability to the local context while still leaving the core elements of the initial project unchanged. This stage is also done in each of the other earlier stages with the refinement process, consultation and collaboration are employed to support agreement about the project steps and its success. Consolidating successful interventions in a dynamic health service is challenging as it requires the use of a number of strategies, adapted to local context, that need to all work in sync. This process needs a few steps as follows; firstly, standardising policies and protocols across the health care setting to support minimal variability across departments; secondly, eliminating variances between policies and practices to support that processes are understood and orderly. Thirdly, having the right staff mix, with the appropriate skills and experience at all times; fourthly, having an idea about expected patient numbers that will benefit from the proposed intervention and ensuring that resources are available to meet any possible increase in numbers [3, 24]. Finally, having access to business intelligence tools such as deidentified patients data on patients care, real time prescribing data and online clinical improvement tools to continue and refine outcomes based on real time numbers is crucial for the success of this step as shown in Table 2.

All the elements discussed above on each of the three stages of the Triple C model have been mentioned by Proctor et al., 2015 to support sustainability of interventions [35]. The authors suggested that for sustainability of evidence-based interventions to occur, various factors need to be included in implementation models and these are training and funding, context, definitions and conceptualization and measurement and analysis. These factors have been captured by the above three stages through having the right skills mix of staff and resources, clarity of responsibilities, process mapping and having access to business intelligence tools respectively as detailed in Table 2 for each of the above stages [35].

### Examples of using the Triple C model in health services research

The Triple C model has been used in several projects to verify its fit for purpose and to support its applicability to implementation science. This is the final stage of building a theory as stated above. The Triple C model was used in the successful implementation of an electronic wound care program across several organisations to track would healing and costs in rural Victoria in Australia [65-70]. The authors were able to show a significant improvement of wound healing times and decreasing dressing [66]. The researchers used the consultation stage to identify priority areas of research and a map of operations for the project delivery. Sufficient resources were made possible by engaging multiple stakeholders early in the project through both in kind and financial resources. The collaboration stage was crucial as this project involved several organisations and training programs to support the successful delivery of the project across the multiple sites. A train-the-trainer program was devised to support efficient implementation of the program. The consolidation stage involved several steps such as standardising policies and procedures across all sites on management of wounds across the rural region and the establishment of a regional wound consultant role to oversee the project after its expiry. These strategies were chosen by the project team to support long-term sustainability of the program. This was in addition to continuous data collection across the sites to promote quality monitoring of wound healing and costs and its consistency with the initial plan of the project [66].

The second project where the Triple C model was used was in the implementation of a medication safety program in an Aboriginal health organisation in a large regional area in Australia [67]. This project employed a three-stage approach. The first stage was consultation where interviews were conducted with staff and Aboriginal health professionals to identify problems with medication issues in the Aboriginal community. The results from these interviews have informed a process map about the intervention to address the needs identified in the consultation stage [68]. The collaboration stage consisted of identifying the staff mix to deliver the intervention. In this case, it was a medication safety program which consisted of staff training and development of policies addressing medication safety to be made available for all staff through an online platform. Embedding the program into staff training and policy were strategies identified by the project team to support sustainability. The consolidation stage of the project involved collation of data regarding satisfaction with the program and medications incidents [67, 68].

Another project where the Triple C model was employed was the development of a skills matrix to identify areas of need to upskill palliative care nurses [69]. Sustainability was considered from the outset of the project as the overall objective was to design and deliver educational programs that are relevant to the needs of palliative care staff across a large rural region involving several organisations. The project started with several consultation sessions addressing the training needs of staff involved in several organisations. Once the needs were identified, a process map regarding the delivery and implementation of the intervention was designed which included the development of a skills matrix to be used by managers for individual staff appraisals and to identify their training needs and their levels of progress throughout the year, as the training occurs. The consolidation stage involved the use of this matrix as a standard form for staff appraisals and discussion about opportunities for future improvements [69].

 A final example of a project using the Triple C model in practice was the implementation of the validated Distress Thermometer to improve identification, assessment and management of distress in the cancer care inpatient wards. This project was the result of a clinical incident and a root cause analysis recommendation. The setting for the project was the private and public inpatient oncology wards at a large tertiary referral hospital. Initially, the project involved consultation with all identified stakeholders to support BUY-in and to identify areas for improvement across the public and private settings. Next, a procedure for management of patient distress in the oncology inpatient setting was developed in collaboration with multidisciplinary teams across oncology including; nurses, doctors and social workers. Staff were involved in the development of strategies that would be used to change practice including initiatives such as regular education sessions on identifying and managing distress for patients admitted to the cancer care inpatient wards and debrief sessions for staff where at-risk patients could be identified. The consolidation stage which considered the sustainability of the implemented interventions involved modifications to existing clinical documentation as well as the availability of a patient information brochure for patients and families to support sustainability of the changes [70, 71].

### Limitation of the model

While this model is adaptable to many contexts as shown above by the variety of projects, its utilisation will be limited by the resources available and time needed for each project. In addition, capacity and capability of staff involved in the implementation will influence successful use of the model. The availability of the business intelligence tools is also a limitation if electronic medical records are not embedded in the hospital system or if there is no method of collecting data automatically as this can be a very laborious task. To address these limitations, project teams need to work closely with organisational leadership to support appropriate resources, education and systems are available.

## Conclusions

This paper has presented the conceptualisation and application of the Triple C model of implementation, highlighting the importance of considering sustainability at all stages of a project. The model is based on theory building in sociology where a literature search identifying barriers and facilitators of complex interventions were mapped followed by a framework design incorporating the findings. The design of the framework was adapted from a sociology theory building concept based on description and explanation of the key concepts involved followed by aligning of the knowledge formed with evidence from the literature. Further elements to the model was added to support its sustainability and these were adaptability to local context and the introduction of business intelligence tools to support its continuous improvement and becoming embedded into practice.

## Data Availability

Not applicable.

## Abbreviations

Triple C: Consultation, Collaboration and Consolidation
